# PinX1 suppresses bladder urothelial carcinoma cell proliferation via the inhibition of telomerase activity and p16/cyclin D1 pathway

**DOI:** 10.1186/1476-4598-12-148

**Published:** 2013-11-23

**Authors:** Jian-Ye Liu, Dong Qian, Li-Ru He, Yong-Hong Li, Yi-Ji Liao, Shi-Juan Mai, Xiao-Peng Tian, Yan-Hui Liu, Jia-Xing Zhang, Hsiang-Fu Kung, Yi-Xin Zeng, Fang-Jian Zhou, Dan Xie

**Affiliations:** 1 State Key Laboratory of Oncology in South China, Sun Yat-Sen University Cancer Center, Collaborative Innovation Center for Cancer Medicine, No. 651, Dongfeng Road East, Guangzhou 510060, China; 2 Department of Urology, Sun Yat-Sen University Cancer Center, No. 651, Dongfeng Road East, Guangzhou 510060, China; 3 Department of Pathology, Sun Yat-Sen University Cancer Center, No. 651, Dongfeng Road East, Guangzhou 510060, China; 4 Department of Pathology, Guangdong Provincial People’s Hospital, No. 106, 2nd Zhongshan Road, Guangzhou 510080, China; 5 Department of Hematology, The First Affiliated Hospital of Sun Yat-Sen University, No. 58, 2nd Zhongshan Road, Guangzhou 510080, China; 6 Department of Radiation Oncology, Tianjin Medical University Cancer Institute & Hospital, Huanhuxi Road, Tiyuanbei, Tianjin 300060, China

**Keywords:** Urothelial carcinoma of bladder, Telomerase activity, p16/cyclin D1 pathway, Prognosis, PinX1

## Abstract

**Background:**

PIN2/TRF1-interacting telomerase inhibitor1 (PinX1) was recently suggested as a putative tumor suppressor in several types of human cancer, based on its binding to and inhibition of telomerase. Moreover, loss of PinX1 has been detected in many human malignancies. However, the possible involvement of PinX1 and its clinical/prognostic significance in urothelial carcinoma of the bladder (UCB) are unclear.

**Methods:**

The PinX1 expression profile was examined by quantitative real-time polymerase chain reaction (qRT-PCR), western blotting, and immunohistochemistry (IHC) in UCB tissues and adjacent normal urothelial bladder epithelial tissues. PinX1 was overexpressed and silenced in UCB cell lines to determine its role in tumorigenesis, development of UCB, and the possible mechanism.

**Results:**

PinX1 expression in UCB was significantly down-regulated at both mRNA and protein level as compared with that in normal urothelial bladder epithelial tissues. PinX1 levels were inversely correlated with tumor multiplicity, advanced N classification, high proliferation index (Ki-67), and poor survival (*P* < 0.05). Moreover, overexpression of PinX1 in UCB cells significantly inhibited cell proliferation in vitro and in vivo, whereas silencing PinX1 dramatically enhanced cell proliferation. Overexpression of PinX1 resulted in G1/S phase arrest and cell growth/proliferation inhibition, while silencing PinX1 led to acceleration of G1/S transition, and cell growth/proliferation promotion by inhibiting/enhancing telomerase activity and via the p16/cyclin D1 pathway.

**Conclusions:**

These findings suggest that down-regulation of PinX1 play an important role in the tumorigenesis and development of UCB and that the expression of PinX1 as detected by IHC is an independent molecular marker in patients with UCB.

## Background

Urothelial carcinoma of the bladder (UCB) is one of the major causes of morbidity and mortality in Western countries [1]. Clinically, radical cystectomy (RC) remains the most common treatment for patients with muscle-invasive UCB or for patients with superficial disease that is at high risk of recurrence and progression. Despite advancement of the surgical technique and the development of novel drugs [2,3], approximately 35% of UCB patients will relapse after treatment, and 5-year cancer-specific survival remains at only 50-60% [4]. It is known that the pathogenesis of UCB is a multistep process that involves multiple genetic changes, including loss of tumor suppressor genes and activation of oncogenes [5]. Although the molecular and/or genetic alterations of UCB have been widely studied, the discovery of specific molecular markers that are present in UCB cells that could serve as reliable clinical/prognostic factors remains substantially limited to date.

PIN2/TRF1-interacting telomerase inhibitor1 (PinX1) is a newly cloned gene mapped to chromosome 8p23.1 that consists of seven exons in humans and is a region frequently associated with loss of heterozygosity in a variety of human malignancies [6-10]. PinX1 has been identified as a critical component in regulating telomerase activity, and is proposed to be a putative tumor suppressor [11]. In humans, ectopic overexpression of PinX1 leads to a decrease in both telomerase activity and cancer cell tumorigenicity, whereas suppression of PinX1 expression results in an increase in both telomerase activity and cancer cell tumorigenicity [11]. Very recently, Chang et al. reported that high significance between a single-nucleotide polymorphism on the PinX1 gene and lower bladder cancer risk [12]. However, the biological function of PinX1 on UCB tumorigenesis and tumor progression has not been characterized. In this study, we investigated the clinicopathological and prognostic significance as well as the potential role of PinX1 in the development and progression of UCB.

## Materials and methods

### Patient information and tissue microarray

To prepare of the bladder tissue microarray (TMA), 187 patients with UCB that had undergone RC were selected from the surgical pathology archives of the Department of Pathology of the Sun Yat-Sen University Cancer Center, the First Affiliated Hospital of Sun Yat-Sen University, and Guangdong Provincial People’s Hospital between 1999 and 2008. The median follow-up time was 92 months (range 8–156 months) and the clinicopathological characteristics are summarized in Table 1. Prior patient consent and approval from the Institutional Research Ethics Committee were obtained for the use of these clinical materials for research purposes. The tumor specimens were obtained from the paraffin blocks of 187 primary UCBs. We also obtained 102 samples, in paraffin blocks, of normal bladder mucosa in adjacent non-neoplastic bladder tissue from the same UCB patients. The TMA was constructed according to a method described previously [13]. In our constructed bladder tissue TMA, three sample cores were selected from each primary UCB and normal bladder tissue. Multiple sections (5-μm thick) were obtained from the TMA block and mounted on microscope slides. Tumor grade and stage were defined according to the criteria of the World Health Organization and the sixth edition of the TNM classification of the International Union Against Cancer (UICC, 2002).

**Table 1:** Correlation of PinX1 expression in tissue with patients’ clinicopathological variables in 187 cases of UCB

		**PinX1 expression (%)**		
**Variables**	**All cases (*N*** **= 187)**	**Negative expression (*N*** **= 83)**	**Positive expression (*N*** **= 104)**	***P *** **value**^**a**^
Age(years)				0.456
≤60^b^	80	33(41.3)	47(58.8)	
>60	107	50(46.7)	57(53.3)	
Gender				0.212
Male	166	71(42.8)	95(57.2)	
Female	21	12(57.1)	9(42.9)	
Tumor multiplicity				<0.001
Unifocal	79	17(21.5)	62(78.5)	
Multifocal	108	66(61.1)	42(38.9)	
WHO grade				0.844
G1	46	19(41.3)	27(58.7)	
G2	66	29(43.9)	37(56.1)	
G3	75	35(46.7)	40(53.3)	
pT status				0.404
pT1	35	13(64.0)	22(36.0)	
pT2	95	38(54.5)	57(45.5)	
pT3	37	20(50.0)	17(50.0)	
pT4	20	12(37.5)	8(62.5)	
pN status				0.023
pN-	157	64(40.8)	93(59.2)	
pN+	30	19(63.3)	11(36.7)	
ki-67 index				0.004
≥50%	95	52(54.7)	43(45.3)	
<50%	92	31(33.7)	61(66.3)	

^a^Chi-square test. ^b^mean age. UCB: urothelial carcinoma of bladder.

#### Immunohistochemistry

Immunohistochemistry (IHC) studies were performed using a standard streptavidin-biotin-peroxidase complex method [14,15]. TMA slides were dried overnight at 37°C, dewaxed in xylene, rehydrated with graded alcohol, and immersed in 3% hydrogen peroxide for 20 min to block endogenous peroxidase activity. Antigen retrieval was carried out in a microwave oven with 10 mM citrate buffer (pH 6.0) for 15 min. The slides were incubated with 10% normal goat serum at room temperature for 10 min to reduce nonspecific reactions. Subsequently, the TMA slides were incubated overnight at 4°C with rabbit polyclonal antibody against PinX1 (1:200; Proteintech Group, USA), mouse monoclonal anti-Ki-67 (1:100; Sigma-Aldrich, USA), or mouse monoclonal anti-p16 (1:100; Cell Signaling Technology, USA) and anti-cyclin D1 (1:100; Cell Signaling Technology, USA), overnight at 4°C. After rinsing five times with 0.01 mol/L phosphate-buffered saline (PBS; pH 7.4) for 10 min, primary antibody was detected using a secondary antibody (Envision; Dako, Glostrup, Denmark) for 1 h at room temperature and stained with 3,3-diaminobenzidine (DAB) after washing in PBS again. Finally, the sections were counterstained with Mayer’s hematoxylin, dehydrated, and mounted.

Two independent pathologists blinded to the clinicopathological information performed the analysis of IHC for PinX1. Similar to that observed in other human tissues [16,17], positive expression of PinX1 in epithelial cells of bladder tissues was primarily in nuclear pattern. PinX1 immunoreactivity was classified into two groups as previously described [17]: negative expression, when PinX1 positive cells were less than 50%; and positive expression, when at least 50% of the cells showed positive staining of PinX1. For the Ki-67 labeling index, the proportion of positive cells in the stained sections was evaluated at × 200 magnification and the mean value of 10 representative fields analyzed from each section was recorded. Previous scoring criterions were used for evaluation of the p16 and cyclin D1 IHC staining [18,19].

### UCB cell lines and cell cultures

The UCB cell lines EJ, T24, and 5637 were cultured in RPMI 1640 (Invitrogen, USA) supplemented with 10% fetal bovine serum (HyClone, USA). All cells were grown in a humidified incubator at 37°C with 5% CO2.

### Paired tumor and adjacent tissues

Ten pairs of UCB tissues and matched adjacent, morphologically normal bladder epithelial tissues were frozen and stored in liquid nitrogen until used to compare the expression levels of PinX1 mRNA and protein.

### RNA extraction and quantitative real-time polymerase chain reaction (qRT-PCR)

Total RNA was isolated from the 10 pairs of UCB tissue and normal bladder tissue using TRIZOL reagent (Invitrogen, USA). RNA was reverse-transcribed using SuperScript First Strand cDNA System (Invitrogen, USA) according to the manufacturer’s instructions. The PinX1 sense primer was 5'-ATGTCTATGCTGGCTGAA-3', and the antisense primer was 5'-TCTGTGGCTCCTTGCT-3'. For the GAPDH gene, the sense primer was 5'- CCCACATGGCCTCCAAGGAGTA -3', and the antisense primer was 5'- GTGTACATGGCAACTGTGAGGAGG -3'. qRT-PCR was done using SYBR Green PCR master mix (Applied Biosystems, USA) in a total volume of 20 μl on the 7900HT fast Real-time PCR system (Applied Biosystems, USA) as follows: 50°C for 2 min, 95°C for 10 min, 40 cycles of 95°C for 15 s, and 60°C for 60 s. A dissociation procedure was performed to generate a melting curve for confirmation of amplification specificity. GAPDH was used as the reference gene. The relative levels of gene expression were represented as ΔCt = Ct_gene_- Ct_reference,_ and the fold change of gene expression was calculated by the 2^-ΔΔCt^ Method. Experiments were repeated in triplicate.

#### Western blotting

Equal amount of whole-cell lysates were resolved with sodium dodecyl sulfate-polyacrylamide gel electrophoresis and transferred to a polyvinylidene difluoride membrane (Pall Corp., USA). This was followed by incubation with primary rabbit polyclonal antibody against human PinX1 (Proteintech Group, USA), mouse monoclonal antibodies to p16 (Cell Signaling Technology, USA), cyclin D1 (Cell Signaling Technology, USA), CDKN2B (Cell Signaling Technology, USA), CCND2 (Cell Signaling Technology, USA), rabbit monoclonal antibodies GADD45A (Santa Cruz Biotechnology, USA), ANAPC2 (Santa Cruz Biotechnology, USA), and CDK5R1 (Santa Cruz Biotechnology, USA), respectively. The immunoreactive proteins were detected with enhanced chemiluminescence detection reagents (Amersham Biosciences, Sweden) according to the manufacturer’s instructions. The membranes were stripped and re-blotted with a mouse monoclonal anti-GAPDH antibody (Santa Cruz Biotechnology, USA) as a loading control.

### Construction of the recombinant lentiviral vector

The PinX1 expression construct was generated by subcloning the PCR-amplified human PinX1 coding sequence into the pBABE retroviral vector. The construction of the PinX1 short hairpin RNA (shRNA) lentiviral expression vector and retroviral production and infection have been described previously [11,17]. Based on their baseline expression of PinX1, UCB cells were transduced with either pBABE/PinX1 or pSUPER-retro-PinX1-shRNA. EJ and T24 cells showed low expression of PinX1 and they were infected with retroviruses carrying pBABE/PinX1. The 5637 cells showed had high expression of PinX1 and they were infected with retroviruses carrying pSUPER-retro-PinX1-shRNA.

### Cell proliferation assay and colony-forming assay

For cell proliferation assays, cells were reseeded in 96-well plates at 2 × 10^3^ cells/well 24 h after transfection and incubated overnight in 100 μL of culture medium. Then, 20 μL of 5 mg/mL 3-(4, 5-dimethylthiazol-2-yl)-2, 5-diphenyltetrazolium bromide (MTT, Sigma-Aldrich, USA) was added to the wells and cells were incubated at 37°C for 4 h. The supernatant was removed, and 150 μL dimethyl sulfoxide was added to the wells. After incubating at 37°C for 15 min, absorbence at 570 nm was measured with a microplate reader (SpectraMax M5, Molecular Devices, USA).

For colony-forming assays, cells were reseeded at 500 or 1000 cells/well in 6-well plates at 24 h after transfection, with medium replacement every three days. After incubating at 37°C for 2–3 weeks, cells were fixed and stained with crystal violet.

#### Flow cytometry

For cell cycle analysis, cells were collected at the indicated time points. Cells (1 × 10^6^) were washed with PBS and fixed with cold 70% ethanol at 4°C overnight. Then, cells were treated with RNase and stained with propidium iodide (PI, Sigma-Aldrich, USA). The DNA content of the cells was quantified using a flow cytometer (Epics Elite, Beckman Coulter, USA). In total, 10,000 nuclei were examined in the flow cytometer, and DNA histograms were analyzed by ModFit software (Verity Software House, USA).

For apoptosis analysis, cells transfected with above mentioned formulations were stained with annexin V-PE and propidium iodide (PI) 48 h post-transfection using the Annexin V apoptosis detection kit (BD biosciences, USA). The percentage of apoptotic cells was quantified by flow cytometry. Viable cells are both Annexin V-PE and PI negative.

### Telomerase activity assay

The telomerase activity was examined when the cells at the 15 passage. Telomerase activity was measured with the TRAPeze telomerase detection kit (Chemicon, USA). PCR products were separated by electrophoresis on a 12.5% nondenaturing polyacrylamide gel, visualized by SYBG green (Invitrogen, USA) staining and semi-quantitated according to the manufacturer’s instruction. Briefly, telomerase activity consists of the intensity of the TRAP product band and the processivity of TRAP ladders.

### Telomere lengths analysis

The telomere length was examined when the cells at the 15 passage. Two micrograms of gemonic DNA from tissue extracts were doubly digested with Hinf I and Rsa I overnight at 37°C. The DNA products of enzymes digestion were electrophoresed on 0.8% agarose gel, and transferred onto a nylon membrane for hybridization with digosin-labbed (TTAGGG)3 oligos. The hybridization signal was detected by the AP-conjugated anti-digosin antibodies (Roche Diagnostics, Indianapolis, Indiana, USA) and imaged by CDP-Star (Roche, Switzerland).

### In vivo tumorigenicity assays

In total, male BALB/c nu/nu immune deficient mice (6 weeks old, 18–20 g) were purchased from Shanghai Slac Laboratory Animal Co., Ltd. (Shanghai, China). The mice were housed in barrier facilities on a 12 h light/dark cycle. All experimental procedures were approved by the Institutional Animal Care and Use Committee of Sun Yat-Sen University. Cells (5 × 10^6^ EJ-Vector, 5 × 10^6^ EJ-PinX1, 5 × 10^6^ T24-Vector, 5 × 10^6^ T24-PinX1, 5 × 10^6^ 5637-Scramble, 5 × 10^6^ 5637-PinX1-shRNA) were suspended in RPMI 1640 medium and injected subcutaneously into the flank of mice. The tumor diameter was measured and the volume (width^2^ × length × 0.52) calculated every other day. Mice were humanely killed on day 48, and the tumors were dissected and weighed.

#### Statistical analysis

Data were analyzed using SPSS16.0 software (SPSS Inc.). Significant associations between PinX1 expression and clinicopathological parameters were assessed using a χ^2^ test. Survival curves were plotted by Kaplan–Meier analysis and compared by the log-rank test. Cox regression analysis was carried out to assess the significance of variables for survival. Data were expressed as mean ± SD, and the *t*-test was used to determine the significance of differences between two groups. All tests carried out were two-sided. *P* < 0.05 was considered statistically significant.

## Results

### qRT-PCR and Western blotting analysis of PinX1 expression in bladder tissues

**Figure 1:**
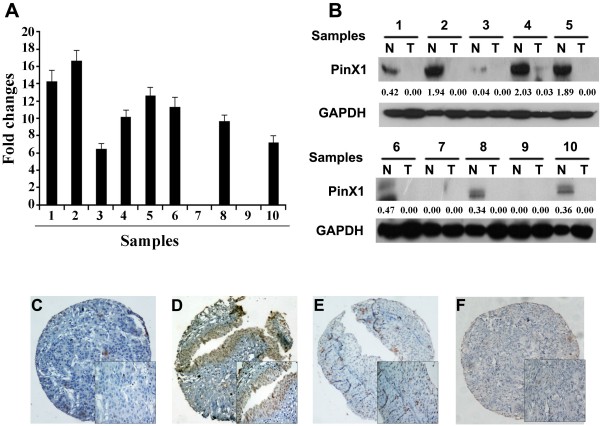
**The expression of PinX1 in UCB and adjacent normal bladder tissues. (A)** Down-regulated expression of PinX1 mRNA was examined by qRT-PCR in 8/10 UCB cases, when compared with adjacent normal bladder tissues. Expression levels were normalized for GAPDH. Error bars, SD calculated from three parallel experiments. **(B)** Down-regulated expression of PinX1 protein was detected by Western blotting in 7/10 UCB cases, when compared with adjacent normal bladder tissues. Expression levels were normalized with GAPDH. **(C-F)** The expression of PinX1 in UCB and adjacent normal bladder tissues by IHC (100×). An UCB (case 45) tissue showed negative expression of PinX1 **(C)**, while its adjacent normal bladder urothelial mucosal tissue was positive stained by PinX1, in which more than 90% of tumor cells were positively stained by PinX1 in the nucleus **(D)**. Negative expression of PinX1 was observed in another UCB tissue (case 73), in which only 10% of tumor cells demonstrated a nuclear staining of PinX1 **(E)**. An UCB (case 126) was negatively stained by PinX1 **(F)**.

Our qRT-PCR results showed that PinX1 mRNA expression was downregulated in eight out of 10 UCB samples compared with the paired normal bladder tissues (Figure 1A). Western blotting analyses also demonstrated downregulation of the PinX1 protein in seven out of 10 UCB samples as compared to their normal counterparts (Figure 1B).

### IHC analysis of PinX1 expression in TMA of bladder tissues

The expression of PinX1 protein was determined by IHC in a TMA containing 187 cases of UCBs and 102 specimens of adjacent normal bladder tissues. Using the criteria described earlier, negative expression of PinX1 was detected in 44.4% (83/187) of UCBs, while only 20.6% (21/102) of normal bladder tissues had negative staining (Figure 1C-1F, *P* = 0.004, χ^2^ test for trend).

### Association of PinX1 expression with UCB clinicopathological features

The association between PinX1 expression in UCB detected by IHC and several known clinicopathological features were studied further. PinX1 levels were inversely correlated with tumor multiplicity and advanced N classification (*P* < 0.05, Table 1). A significant correlation between the Ki-67 labeling index and PinX1 expression in UCB was also found (*P* = 0.004, Table 1). There was no significant association between PinX1 expression and other clinicopathological features, such as patient gender, age, tumor grade, and pT classification (*P* > 0.05, Table 1).

### Relationship between clinicopathological variables, PinX1 expression, and UCB patient survival: univariate survival analysis

**Figure 2:**
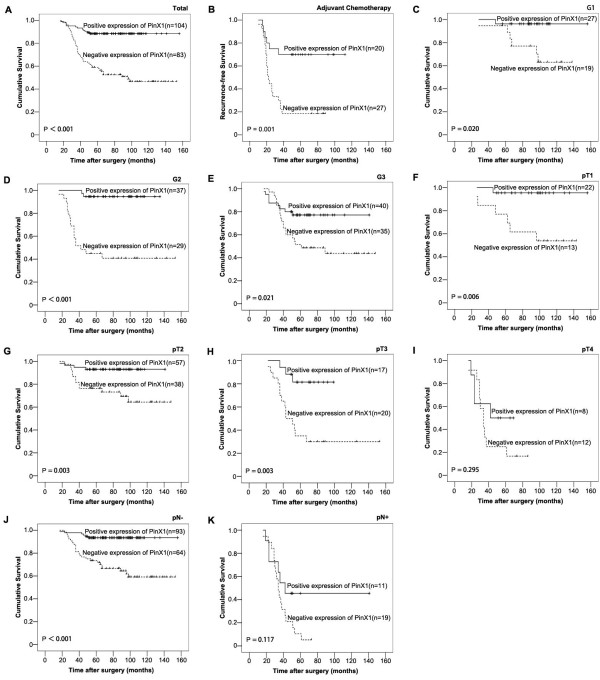
**Kaplan-Meier survival analysis of PinX1 expression in patients with UCB (log-rank test). (A)***Total,* probability of overall survival of all patients with UCB: negative expression (*dashed line*), *n* = 83; positive expression (*solid line*), *n* = 104. **(B)***Adjuvant chemotherapy,* probability of recurrence-free survival of patients underwent adjuvant chemotherapy with UCB: negative expression (*dashed line*), *n* = 27; positive expression (*solid line*), *n* = 20. **(C)***G1,* probability of overall survival of G1 patients with UCB: negative expression (*dashed line*), *n* = 19; positive expression (*solid line*), *n* = 27. **(D)***G2,* probability of overall survival of G2 patients with UCB: negative expression (*dashed line*), *n* = 29; positive expression (*solid line*), *n* = 37. **(E)***G3,* probability of overall survival of G3 patients with UCB: negative expression (*dashed line*), *n* = 35; positive expression (*solid line*), *n* = 40. **(F)***pT1,* probability of overall survival of pT1 patients with UCB: negative expression (*dashed line*), *n* = 13; positive expression (*solid line*), *n* = 22. **(G)***pT2,* probability of overall survival of pT2 patients with UCB: negative expression (*dashed line*), *n* = 38; positive expression (*solid line*), *n* = 57. **(H)***pT3,* probability of overall survival of pT3 patients with UCB: negative expression (*dashed line*), *n* = 20; positive expression (*solid line*), *n* = 17. **(I)***pT4-,* probability of overall survival of pT4 patients with UCB: negative expression (*dashed line*), *n* = 12; positive expression (*solid line*), *n* = 8. **(J)***pN-,* probability of overall survival of pN- patients with UCB: negative expression (*dashed line*), *n* = 64; positive expression (*solid line*), *n* = 93. **(K)***pN+,* probability of overall survival of pN+ patients with UCB: negative expression (*dashed line*), *n* = 19; positive expression (*solid line*), *n* = 11.

First, to confirm the representativeness of the UCB in our study, we analyzed the established prognostic predictors of survival in our cohort. Kaplan–Meier analysis demonstrated a significant impact of well-known clinicopathological prognostic parameters on patient survival, such as tumor grade, pT status and pN status (*P* < 0.05, Table 2). Assessment of survival in total UCBs determined that the positive expression of PinX1 was correlated with superior survival (*P* < 0.001, Table 2, Figure 2A).

**Table 2:** Univariate analysis of factors associated with overall survival of 187 patients with UCB

**Variable**	**All cases**	**RR (95% CI)**	***P *** **value**^**a**^	
Age(years)			0.309	
≤60^b^	80	1		
>60	107	1.332 (0.767-2.315)		
Gender			0.648	
Male	166	1		
Female	21	0.807 (0.321-2.026)		
Tumor multiplicity			0.676	
Unifocal	79	1		
Multifocal	108	1.443 (0.852-2.471)		
WHO grade			0.022	
G1	46	1		
G2	66	2.304 (0.968-5.485)		
G3	75	3.206 (1.396-7.363)		
pT status			<0.001	
pT1	35	1		
pT2	95	1.535 (0.890-2.653)		
pT3	37	3.025 (1.242-7.363)		
pT4	20	7.457 (2.950-18.847)		
pN status			<0.001	
pN-	157	1		
pN+	30	8.904 (5.056-15.681)		
PinX1			<0.001	
Negative expression	83	5.148 (2.708-9.786)		
Positive expression	104	1		

^a^Chi-square test. ^b^mean age. RR: relative risk. CI: confidence interval.

UCB: urothelial carcinoma of bladder.

Moreover, we analyzed the recurrence-free survival of patients who received adjuvant chemotherapy. Interestingly, we found that patients with negative PinX1 expression had a much higher risk of recurrence than did patients with positive PinX1 expression. As shown in Figure 2B, the 5-years recurrence-free survival rate was only 19.0% in the PinX1-negative group, whereas it dramatically increased to 70.0% in the PinX1- positive group (log-rank test, *P* = 0.001, Figure 2B).

Furthermore, stratified survival analysis determined that PinX1 expression could differentiate the survival of the UCB patients with grades 1, 2, and 3 tumors (*P* = 0.020, < 0.001, and 0.021, respectively, Figure 2), as well as with pT1 (*P* = 0.006), pT2 (*P* = 0.003), pT3 (*P* = 0.003), and pN- (*P* < 0.001) classifications (Figure 2).

### Independent prognostic factors of UCB: multivariate Cox regression analysis

The expression of PinX1 as well as other clinical pathological parameters that were significant in univariate analysis (grade, pT stage, pN stage), was further examined in multivariate analysis. Negative expression of PinX1 was found to be an independent prognostic factor for poor overall survival (relative risk: 4.122, 95% confidence interval: 2.152–7.896, *P* < 0.001, Table 3). Of the other parameters, pT stage, and pN stage were also demonstrated as independent prognostic factors for overall survival (*P* < 0.05, Table 3).

**Table 3:** Multivariate analysis of prognostic factors on overall survival (Cox regression model)

**Variable**	**Hazards ratio**	**95% CI**	***P *** **value**
WHO grade ( G1 vs G2 vs G3)	1.045	0.697-1.566	0.831
pT status ( pT1 vs pT2 vs pT3 vs pT4)	2.483	1.032-5.981	0.042
pN status ( pN- vs pN+)	7.169	4.021-12.783	<0.001
PinX1 ( Positive vs Negative)	4.122	2.152-7.896	<0.001

CI: confidence interval.

### PinX1 inhibits proliferation and clonogenicity of UCB cells

**Figure 3:**
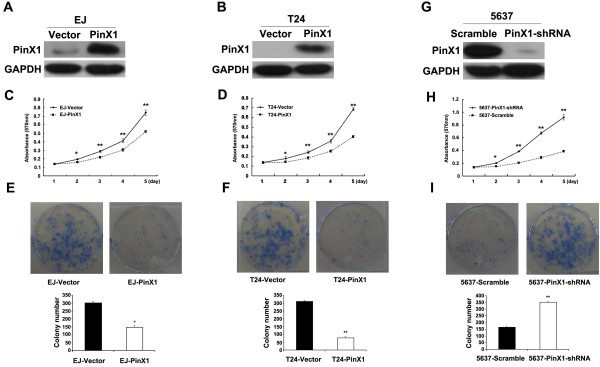
**PinX1 inhibited growth and proliferation of UCB cells in vitro. (A-B)** Ectopic expression of PinX1 in EJ and T24 cell analyzed by western blotting. GAPDH was used as a loading control. **(C-D)** Ectopic expression of PinX1 inhibited EJ and T24 cell proliferation in MTT assays. Each bar represents the average ± SD of three independent experiments. **(E-F)** Upregulation of PinX1 inhibited EJ and T24 cell growth in colony formation assays. Representative micrographs (upper) and quantification (lower) of crystal violet-stained cells. **(G)** RNAi-silencing of PinX1 in shRNA-transduced stable 5637 cell. GAPDH was used as a loading control. **(H)** Silencing endogenous PinX1 promoted cell growth as determined by MTT assays. Each bar represents the average ± SD of three independent experiments. **(I)** Downregulation of PinX1 promoted 5637 cell growth in colony formation assays. Representative micrographs (upper) and quantification (lower) of crystal violet-stained cells. ^*^P < 0.05, ^**^P < 0.01.

The stable PinX1-expressing cell lines EJ-PinX1 and T24-PinX1 were established (Figure 3A and 3B) to study the biological role of PinX1 in UCB growth/proliferation. Western blotting revealed that PinX1 protein was highly expressed in the EJ-PinX1 and T24-PinX1 cells, whereas expression low or not detected in the stable EJ-Vector and T24-Vector control cell lines, respectively. In the MTT assay, EJ-PinX1 and T24-PinX1 cells grew more slowly, with 1.4-fold and 1.7-fold fewer cells than the EJ-Vector and T24-Vector control cells respectively, by day 5 after plating (Figure 3C and 3D). In the colony-formation assay, EJ-PinX1 and T24-PinX1 cells also formed fewer and smaller colonies than the EJ-Vector and T24-Vector cells, respectively. (Figure 3E and 3F).

Furthermore, knocking-down of endogenous PinX1 in 5637 cells by shRNA significantly decreased PinX1 protein expression (Figure 3G) and increased 5637 cell viability, as analyzed by the MTT and colony-formation assays (Figure 3H and 3I).

### PinX1 inhibits xenografted tumor growth in vivo

**Figure 4:**
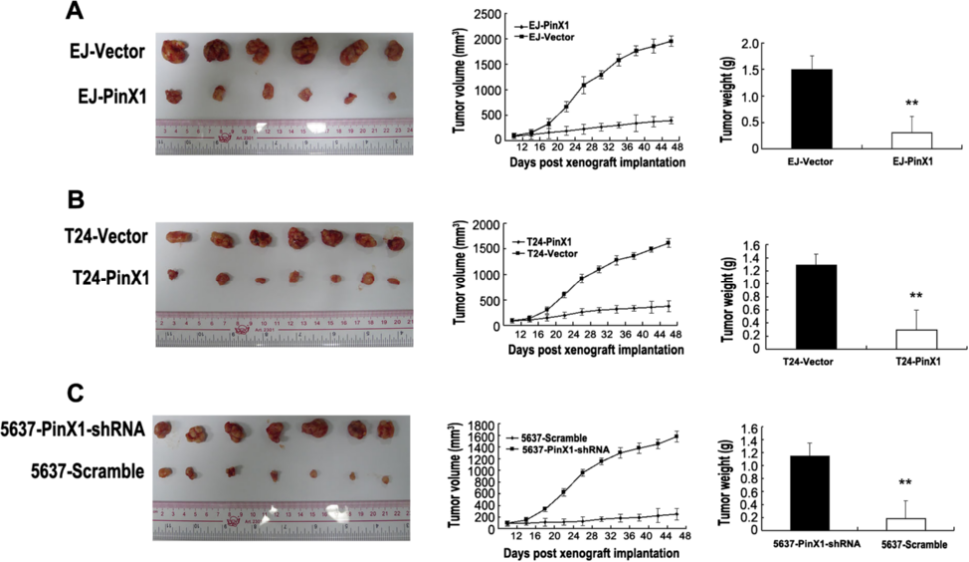
**PinX1 inhibited growth and proliferation of UCB cells in vivo. (A-B)** Ectopic expression of PinX1 in EJ and T24 cells dramatically inhibited tumor growth and proliferation in vivo as determined by a subcutaneous xenograft mice model. Representative graph of tumor growth (left). Data points are the mean tumor volumes ± SD (middle) and mean tumor weights (right) 48 days after inoculation. **(C)** Suppression of PinX1 in 5637 cells promoted tumor growth and proliferation in vivo as determined by a subcutaneous xenograft mice model. Representative graph of tumor growth (left). Data points are the mean tumor volumes ± SD (middle) and mean tumor weights (right) 48 days after inoculation. ^**^P < 0.01.

Tumors formed from EJ-PinX1 and T24-PinX1 cells implanted in nude mice grew more slowly and weighed substantially less than those formed by EJ-Vector and T24-Vector cells respectively, after 48 days (Figure 4A and 4B).

Furthermore, tumors derived from 5637 cells transduced with retroviruses expressing PinX1-shRNA grew much faster and weighed significantly more at day 48 than those formed by 5637-Scramble cells (Figure 4C).

### Effect of PinX1 on UCB cell apoptosis measured by flow cytometry

Cellular apoptosis was examined by the Annexin-V/PI method in UCB cells. Annexin V binds to those cells that express phosphatidylserine on the outer layer of the cell membrane, which is a characteristic feature of cells entering the process of apoptosis. Apoptosis was then quantified by the method of flow cytometry. The incidences of apoptotic death in EJ and T24 cells were increased by the upregulated expression of PinX1 (Additional file 1: Figure S1A and Figure S1B).

Conversely, PinX1 silencing decreased the incidence of apoptotic death in 5637 cells (Additional file 1: Figure S1C).

### Effect of PinX1 on telomerase activity and telomere length in UCB cells

**Figure 5:**
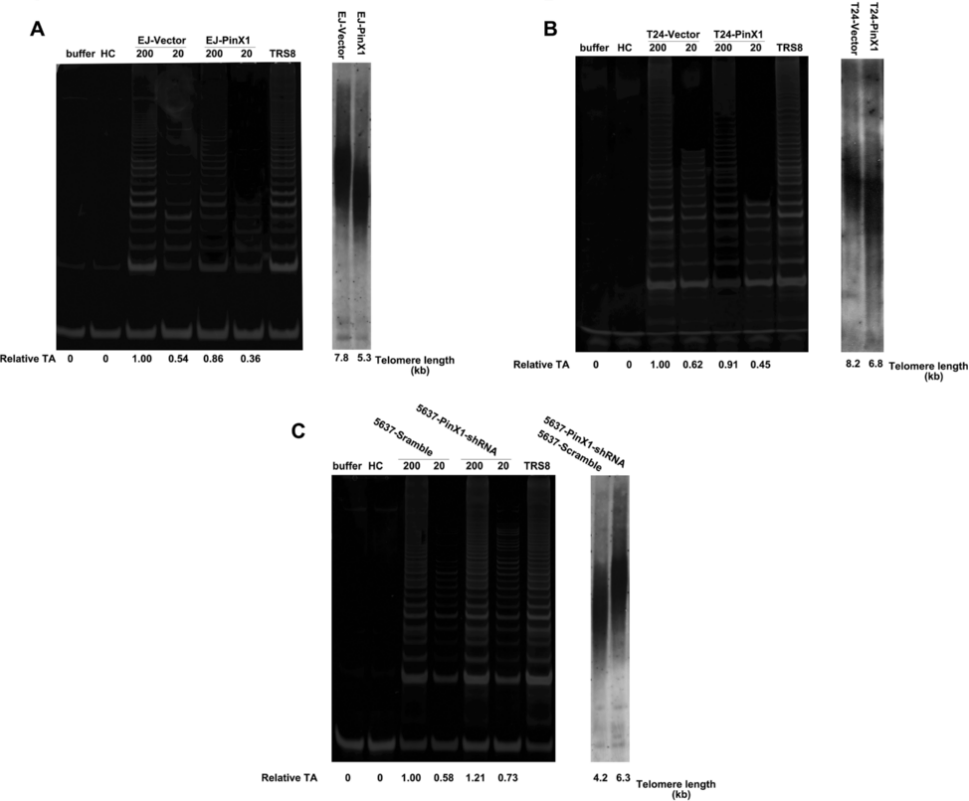
**Effect of PinX1 on telomerase activity and telomere length in UCB cells.** Telomerase activity was measured by TRAP assays and Southern blot analysis of telomeric terminal restriction fragments were used for determination of the telomere length. **(A)** Overexpression of PinX1 in EJ cells decreased telomerase activity and shortened telomere length. **(B)** Overexpression of PinX1 in T24 cells decreased telomerase activity and shortened telomere length. **(C)** RNAi-silencing of PinX1 in 5637 cells increased telomerase activity and elongated telomere length.

As it has been documented that PinX1 could inhibit telomerase activity, shorten telomeres, and suppress tumor growth [20,21], we investigated whether PinX1 could influence tumor growth by regulating telomerase activity and the telomere length pathway. Indeed, we found that overexpression of PinX1 decreased telomerase activity and shortened telomeres in EJ and T24 cells (Figure 5A and 5B).

By contrast, reduced PinX1 by PinX1-shRNA transfection increased telomerase activity and elongated telomere length in 5637 cells (Figure 5C).

### PinX1 regulates G1/S phase transition of the cell cycle

**Figure 6:**
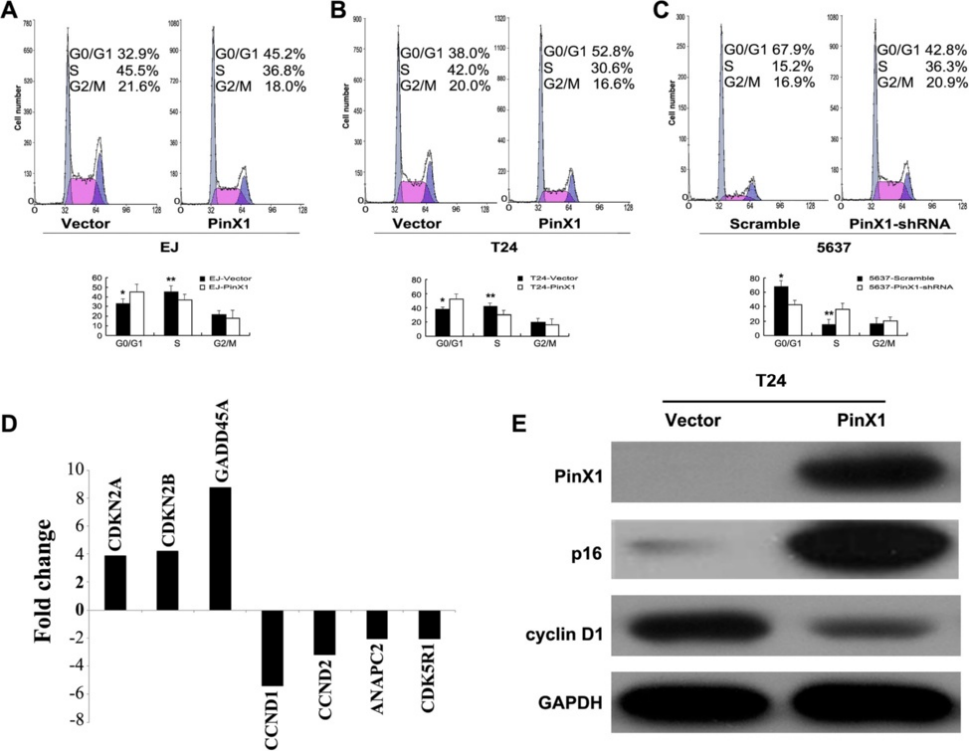
**PinX1 regulates the G1/S phase transition and cell proliferation through the p16/cyclin D1 pathway in UCB cells.** Flow cytometric analysis of the **(A)** EJ bladder urothelial carcinoma cells infected with vector or PinX1, **(B)** T24 bladder urothelial carcinoma cells infected with vector or PinX1, and **(C)** 5637 cells infected with RNAi-vector or PinX1-shRNA. **(D)** The seven genes, CDKN2A (i.e. p16), CDKN2B (i.e. p15), GADD45A, CCND1 (i.e. cyclin D1), CCND2 (i.e. cyclin D2), ANAPC2 and CDK5R1), were examined >2-fold mRNA differential expression in PinX1-transfected T24 cells compared with that of scramble vector transfected by using a Human Cell Cycle RT^2^ Profiler^CC^ PCR Array. **(E)** Overexpression of PinX1 substantially upregulated p16 expression and downregulated cyclin D1 expression in T24 cells detected by western blotting. ^*^P < 0.05, ^**^P < 0.01.

Upregulation of PinX1 expression in EJ and T24 cells significantly increased the proportion of cells in the G0/G1 phase and decreased those in the S phase (Figure 6A and 6B).

Conversely, downregulation of PinX1 in 5637 cells clearly decreased the proportion of cells in the G0/G1 phase and increased those in the S phase (Figure 6C). These findings indicate that PinX1 may play an important role in regulating G1 to S phase transition in UCB cells.

### PinX1 regulated p16 and cyclin D1 expression in UCB cells

To gain further insight into the functions of PinX1 in UCB cell growth and development, the mRNA expression profiles of T24-PinX1 cells were compared with that of T24-Vector using a Human Cell Cycle RT^2^ Profiler^CC^ PCR Array containing 84 cell cycle related genes. The results showed that a total of six up-regulated and five down-regulated genes (> 1.5-fold) were identified in T24-PinX1 cells compared with that in T24-Vector cells (Additional file 2: Table S1). Subsequently, CDKN2A (i.e. p16), CDKN2B (i.e. p15), GADD45A, CCND1 (i.e. cyclin D1), CCND2 (i.e. cyclin D2), ANAPC2, and CDK5R1, which exhibited > 2-fold mRNA differences before and after PinX1 overexpressed (Figure 6D), were selected and further analyzed by western blotting. Consistent with that of mRNA expression in real-time PCR array, increased protein expression of p16 and decreased protein expression of cyclin D1 were examined by western blotting in T24 cells after PinX1 overexpressed (Figure 6E).

### Expression of p16 and cyclin D1 in UCB tissues and their correlation with PinX1 expression

Utilizing the previous scoring criterions for IHC staining evaluation of p16 and cyclin D1 [18,19], there was positive expression of p16 and cyclin D1 in 90/187 (48.1%) and 102/187 (54.5%) of UCBs, respectively. In addition, a significant correlation between the expression of PinX1 and p16 was evaluated in our UCB cohort, in which the frequency of cases with negative PinX1 expression was significantly higher in negative p16 expression cases (54/97 cases, 55.7%) than in positive p16 expression ones (29/90 cases, 32.2%) (*P* = 0.001, Additional file 3: Table S2). A significant correlation between the expression of PinX1 and cyclin D1 was also observed in the UCB tissues (P < 0.001, Additional file 3: Table S2).

## Discussion

It has been proposed that the PinX1 gene could be a putative tumor suppressor gene and/or therapeutic target for human cancers [11,20,21]. Although the relationship between the PinX1 gene and human tumors has been studied widely, such as in medulloblastoma, hepatocelllular carcinoma, prostate cancer, and gastric cancer [15,22-25], the expression and prognostic value of PinX1 protein has not been investigated in UCB. In addition, the molecular mechanisms underlying the potential role of PinX1 in UCB remain unknown. In this study, we examined the expression dynamics status of PinX1 firstly by IHC using a TMA containing a series of UCB and adjacent morphologically normal bladder epithelial tissues. The IHC results demonstrated that negative expression of PinX1 protein in 44.4% of primary bladder tumor, but in only 20.6% of normal bladder epithelial tissues. In addition, western blotting revealed downregulated expression of PinX1 in the majority of UCBs when compared with their adjacent normal bladder epithelial tissues. Furthermore, forced expression of PinX1 in UCB cell lines led to the inhibition of cell proliferation and tumourigenicity in vitro and in vivo, accompanied with G1/S phase arrest, upregulation of p16 expression, downregulation of cyclin D1 expression, as well as the deactivation of telomerase activity. Meanwhile, RNA interference silencing of PinX1 expression induced opposite results. These findings provide evidence for the concept that downregulating the expression of PinX1 may play an important role in the tumorigenic process of UCB.

Further correlation analyses demonstrated that negative expression of PinX1 in our UCB cohort was significantly associated with advanced N classification, higher proliferation index, and tumor multiplicity. Importantly, we found that decreased or depleted expression of PinX1 was associated with poor prognosis and reduced survival periods for UCB patients. Multivariate analysis showed that the loss of PinX1 protein expression could be used as an independent prognostic predictor for UCB patients. Furthermore, in stratified survival analysis, PinX1 expression could differentiate the survival of certain subsets of UCB patients, including patients with grade 1, 2 and 3 tumors and at pT1, pT2, pT3, and pN- stage. Our results indicate that the expression level of PinX1 protein might provide useful information in the evaluation prognosis and follow-up schedule guiding for UCB patients.

PinX1 is an evolutionarily conserved nuclear protein that has been demonstrated to be a telomerase/telomere-interacting factor in humans. Originally, PinX1 was identified as an intrinsic telomerase inhibitor and a putative tumor suppressor because of its binding to and inhibition of telomerase [20]. Recently, it has been reported that human PinX1 can regulate telomerase activity and suppress tumor growth both in vivo and in vitro [20,21]. Overexpression of PinX1 in tumor cells could inhibit telomerase activity, shorten telomeres, and suppress tumor growth, while depletion of endogenous PinX1 increased telomerase activity, elongated telomeres, and enhanced tumorigenicity in telomerase-positive HT1080 cancer cells [20]. Disruption of the PinX1-dependent telomere maintenance pathway could reduce carcinogenesis and enhance chemotherapeutic sensitivity in telomerase-positive human cancer cells as well [11]. In the present study, we found that overexpression of PinX1 by transfection of pBABE-PinX1 into EJ and T24 cells significantly reduced cell growth, and arrested cells in the G0/G1 phase via the inhibition of telomerase activity and shortening of telomeres. In contrast, inhibition of PinX1 expression by shRNA transfection in 5637 cells promoted cell growth/proliferation in vitro and vivo via by enhancing telomerase activity and telomere elongating. These findings suggest that PinX1 acts as an intrinsic telomerase inhibitor and arrests cell growth in human UCB.

We showed that PinX1 could prohibit G1/S phase transition, to gain further insight into the downstream molecular events involving PinX1 and UCB growth/proliferation, we compared mRNA expression profiles between T24-PinX1 and T24-Vector cells using a Human Cell Cycle real-time PCR array containing 84 well-known cell cycle related genes. Of the 84 genes, 7 genes were differentially expressed by 2-fold or more (i.e. upregulated: CDKN2A (i.e. p16), CDKN2B (i.e. p15) and GADD45A; downregulated: CCND1 (i.e. cyclin D1), CCND2 (i.e. cyclin D2), ANAPC2 and CDK5R1). Subsequently, protein expression of these seven genes was analyzed by western blotting. Consistent with that of mRNA expression in the real-time PCR array, upregulated p16 expression and downregulated cyclin D1 expression were validated in the protein level following PinX1 overexpression in T24 cells. It was appear that PinX1 regulated the cell cycle and influenced cell growth/proliferation via the regulation of p16 and cyclin D1 expression in the UCB cells we used. Further, the status of p16 and cyclin D1 expression was examined by IHC in a TMA of a large cohort of UCBs. Our analysis demonstrated that there were significant positive correlations between the expression of PinX1 and p16 and between the expression of PinX1 and cyclin D1, which confirmed the results observed in the T24 cells.

The p16 protein acts as an inhibitor of cell proliferation by competitively binding the cyclin-dependent kinase (CDK)4/6 kinases against their regulator cyclin D1 and blocking phosphorylation of the retinoblastoma (Rb) protein, leading to cell cycle arrest [26]. The p16/cyclin D1 pathway is one of the key signal transduction pathways at the G1/S checkpoint in the cell cycle [27,28]. Dysfunction of the proteins involved in the p16 pathway such as deletion of the p16 gene and overexpression of CDKs of cyclin D1 will lead to Rb phosphorylation, subsequent progression of G1/S phase transition and promotion of uncontrolled cell growth/proliferation [29-34]. Song et al. reported that the decrease of p16 cooperated with cyclin D1 and the caused deregulation of G1/S checkpoint, leading to abnormal cell proliferation in nasopharyngeal carcinoma [35]. These observations, together with the results of our PinX1 functional studies in the UCB cells, suggest that decreased expression of PinX1 in UCB might be involved in the p16/cyclin D1 associated pathway and thus support cancer cell growth/proliferation. Clearly, better understanding of the precise molecular mechanisms of p16 and cyclin D1 regulated by PinX1 may lead to more effective management of UCB growth and/or progression.

Based on previous studies [20,21,26-28,35] and the present study, we propose that PinX1 regulates UCB cell proliferation through at least two distinct mechanisms. In one mechanism, PinX1 influences UCB cell growth/proliferation by binding to telomerase and inhibiting its activity. In the other mechanism, PinX1 inhibites UCB cell growth/proliferation by regulating the expression of the key cell cycle genes for p16 and cyclin D1. More studies are needed to confirm these two mechanisms and to elucidate whether other signaling pathways also contribute to PinX1-mediated cell growth/proliferation in UCB.

In summary, we describe for the first time in this study the protein expression pattern of PinX1 in UCB and adjacent morphologically normal bladder epithelial tissues. Our results provide a basis for the concept that negative expression of PinX1 in UCB may be important in the acquisition of an aggressive and/or poor prognostic phenotype. In addition, the functional studies of PinX1 in this report suggest a potential important role of PinX1 in the control of cell growth/proliferation via the regulation of telomerase activity and the p16/cyclin D1 pathway, an activity that might be responsible, at least in part, for the development and/or ultimately the progression of human UCB.

## Abbreviations

PinX1: PIN2/TRF1-interacting telomerase inhibitor1; UCB: Urothelial carcinoma of the bladder; qRT-PCR: Quantitative real-time polymerase chain reaction; RC: Radical cystectomy; TMA: Tissue microarray; IHC: Immunohistochemistry; PI: Propidium iodide; CDK: Cyclin-dependent kinase; RB: Retinoblastoma.

## Competing interest

The authors declare that they have no competing interests.

## Authors’ contributions

JYL evaluated the clinical records, carried out the experimental work and drafted the manuscript. DQ, LRH, and YHL contributed for data interpretation and drafted the manuscript. XPT critically revised the manuscript. YJL participated in the statistical analysis and help to draft the manuscript. SJM, YHL, and JXZ help to carry out the immunohistochemistry assays. HFK contributed for critical revision of statistical analysis and of the manuscript. FJZ designed the study and participated in its coordination. YXZ and DX participated in the design of the study, in its analysis and in the interpretation of the data. DX also participated in evaluated the immunohistochemistry results and wrote the manuscript. All authors read and approved the final manuscript.

## Supplementary Material

Additional file 1: Figure S1 PinX1 promoted apoptosis of UCB cells. (A) Ectopic expression of PinX1 promoted EJ cell apoptosis by Annexin-V/PI method (*P* = 0.012). (B) Ectopic expression of PinX1 promoted T24 cell apoptosis by Annexin-V/PI method (*P* = 0.013). (C) Sliencing endogenous PinX1 inhibited 5637 cell apoptosis by Annexin-V/PI method (*P* = 0.005). Click here for file

Additional file 2: Table S1 List of genes differentially expressed in T24 cells after PinX1 overexpression using a Human Cell Cycle Real-time PCR Array. Click here for file

Additional file 3: Table S2 Association between expression of PinX1 and p16 and cyclin D1 in UCB. Click here for file
