# Supplementary figures and images for: PinX1 suppresses bladder urothelial carcinoma cell proliferation via the inhibition of telomerase activity and p16/cyclin D1 pathway
Source: Mol Cancer. 2013 Nov 23;12:148. doi: 10.1186/1476-4598-12-148 (PMC4176126; doi:10.1186/1476-4598-12-148)

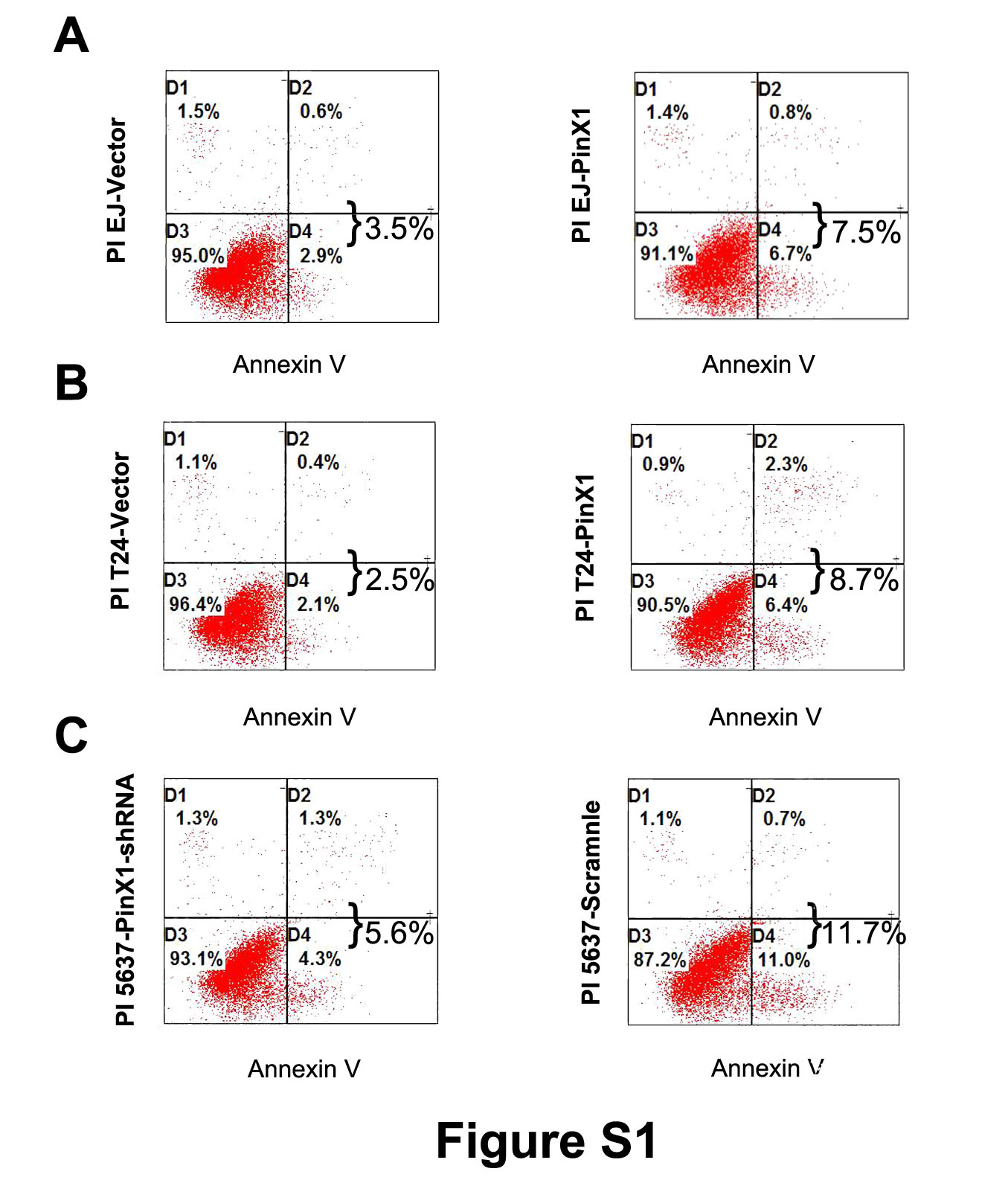

Supplement: Additional file 1: Figure S1 — PinX1 promoted apoptosis of UCB cells. (A) Ectopic expression of PinX1 promoted EJ cell apoptosis by Annexin-V/PI method (P = 0.012). (B) Ectopic expression of PinX1 promoted T24 cell apoptosis by Annexin-V/PI method (P = 0.013). (C) Sliencing endogenous PinX1 inhibited 5637 cell apoptosis by Annexin-V/PI method (P = 0.005). [file 1476-4598-12-148-S1.jpeg]
