# Supplementary material for: PinX1 suppresses bladder urothelial carcinoma cell proliferation via the inhibition of telomerase activity and p16/cyclin D1 pathway
Source: Mol Cancer. 2013 Nov 23;12:148. doi: 10.1186/1476-4598-12-148 (PMC4176126; doi:10.1186/1476-4598-12-148)
Supplement: Additional file 2: Table S1 — List of genes differentially expressed in T24 cells after PinX1 overexpression using a Human Cell Cycle Real-time PCR Array. [file 1476-4598-12-148-S2.doc]

| **Table S1** List of genes differentially expressed in T24 cells after PinX1 overexpression using a Human Cell Cycle Real-time PCR Array | | | |
| --- | --- | --- | --- |
| Gene | Fold change | Location | Function |
| Upregulated genes |  |  |  |
| ACTB | 1.02 | 7p22.1 | ORM cytoskeleton |
| ATM | 1.04 | 11q22.3 | negative regulate cell cycle, respond to DNA damage |
| BAX | 1.25 | 19q13.33 | induction of apoptosis |
| BRCA1 | 1.22 | 17q21.31 | negative regulate cell cycle,  participate DNA repair |
| BRCA2 | 1.19 | 13q13.1 | cell cycle checkpoint and cell cycle arrest |
| CDC20 | 1.16 | 1p34.2 | promote mitosis |
| CDK1B | 1.91 | 12p13.1 | inbibit cell proliferation, promote apotosis |
| CDK5RAP1 | 1.19 | 20q11.21 | suppress cell proliferation and differentiation |
| CDKN1A | 1.56 | 10q26.2 | inbibit cell proliferation, promote apotosis |
| CDKN2A | 3.85 | 9p21.3 | inbibit cell proliferation, promote apotosis |
| CDKN2B | 4.18 | 9p21 | inbibit cell proliferation, inhibit cell cycle progression |
| CDKN3 | 1.07 | 14q22.2 | inbibit cell proliferation, inhibit cell cycle progression |
| CHEK1 | 1.05 | 11q24.2 | inbibit cell proliferation, participate DNA repair |
| CHEK2 | 1.08 | 22q12.1 | prevent cell cycle progression,  response to DNA damage |
| CUL1 | 1.09 | 7q36.1 | inbibit cell proliferation, promote apotosis,  prevent cell cycle progression |
| CUL2 | 1.01 | 10p11.21 | inbibit cell proliferation, promote apotosis,  prevent cell cycle progression |
| CUL3 | 1.07 | 2q36.2 | promote apotosis, prevent cell cycle progression |
| DIRAS3 | 1.25 | 1p31.3 | inhibit growth |
| GADD45A | 8.75 | 1p31.3 | prevent cell cycle progression, promote apotosis,  participate DNA repair |
| GTSE1 | 1.13 | 22q13.2-q13.3 | prevent cell cycle progression, respond to DNA damage |
| HPRT1 | 1.11 | Xq26-q27.2 | nucleotide metabolism |
| KNTC1 | 1.10 | 12q24.31 | participate cell cycle and mitosis |
| KPNA2 | 1.06 | 17q24.2 | participate cell cycle and DNA metabolism |
| MCM3 | 1.11 | 6p12.2 | participate cell cycel, DNA replication,  and cell division |
| MCM4 | 1.07 | 8q11.21 | participate DNA replication |
| MCM5 | 1.12 | 22q12.3 | regulate cell cycle progression,  and DNA replication |
| NBN | 1.04 | 8q21.3 | prevent cell cycle progression,  respond to DNA damage |
| RAD1 | 1.00 | 5p13.2 | participate cell cycle and DNA repair |
| RAD9A | 1.25 | 11q13.2 | regulate cell cycle progression, and DNA repair |
| RAD17 | 1.03 | 5q13.2 | participate cell cycle, DNA repair and replication |
| RAD51 | 1.33 | 15q15.1 | participate DNA repair |
| RB1 | 1.65 | 13q14.2 | prevent cell cycle progression, and cell growth |
| RBL1 | 1.32 | 20q11.23 | prevent cell cycle progression |
| RBL2 | 1.06 | 16q12.2 | prevent cell cycle progression |
| SKP2 | 1.02 | 5p13.2 | prevent cell cycle progression,  and cell proliferation |
| SUMO1 | 1.04 | 2q33.1 | nuclear transport, transcriptional regulation,  apoptosis, and protein stability |
| TP53 | 1.02 | 17p13.1 | inhibit cell cycle, and cell proliferation,  promote apoptosis, senescence |
| Downregulated  genes |  |  |  |
| ABL1 | -1.29 | 9q34.12 | participate cell differentiation, division, adhesion,  and DNA replication |
| ANAPC2 | -2.06 | 9q34.3 | promote progression cell cycle |
| ANAPC4 | -1.41 | 4p15.2 | promote progression cell cycle |
| ATR | -1.30 | 3q23 | promote progression cell cycle, DNA repair |
| B2M | -1.03 | 15q21.1 | immune response, MHC I receptor |
| BCCIP | -1.31 | 10q26.2 | promote progression cell cycle,  resolve spontaneous DNA damage |
| BCL2 | -1.06 | 18q21.33 | inhibit apoptosis |
| BIRC5 | -1.08 | 17q25.3 | inhibit apoptosis |
| CCNB1 | -1.05 | 5q13.2 | promote progression cell cycle |
| CCNB2 | -1.33 | 12q14-q15 | promote progression cell cycle |
| CCNC | -1.12 | 6q16.2 | promote progression cell cycle |
| CCND1 | -5.43 | 11q13.3 | promote progression the cell cycle,  participate metabolism, and cellular differentiation |
| CCND2 | -3.21 | 12p13.32 | promote progression cell cycle |
| CCNE1 | -1.22 | 19q12 | promote cell proliferation |
| CCNF | -1.00 | 16p13.3 | regulate cell cycle, participate M phase |
| CCNG1 | -1.38 | 5q34 | promote proliferation, DNA damage repair |
| CCNG2 | -1.39 | 4q21.1 | promote proliferation, DNA damage repair |
| CCNH | -1.79 | 5q14.3 | promote progression cell cycle,  processes of transcription, DNA repair |
| CCNT1 | -1.25 | 12q13.11-q13.12 | regulate cell cycle, participate G2 phase and G2/M transition |
| CCNT2 | -1.11 | 2q21.3 | regulate cell cycle, participate G2 phase  and G2/M transition |
| CDC16 | -1.09 | 11q11-q17 | promote progression cell cycle |
| CDC34 | -1.18 | 19p13.3 | cell cycle checkpoint and cell cycle arrest |
| CDK1 | -1.09 | 10q21.2 | promote progression cell cycle |
| CDK2 | -1.14 | 12q13.2 | promote cell proliferation |
| CDK4 | -1.23 | 12q14.1 | promote cell proliferation |
| CDK5R1 | -2.05 | 17q11.2 | promote cell proliferation |
| CDK6 | -1.27 | 7q21.2 | promote cell proliferation |
| CDK7 | -1.11 | 5q13.2 | promote cell proliferation |
| CDK8 | -1.11 | 13q12.13 | promote progression cell cycle,  promote cell proliferation |
| CKS1B | -1.12 | 1q21.3 | promote mitosis, promote cell cycle progression |
| CKS2 | -1.31 | 9q22 | promote mitosis, promote cell cycle progression |
| DDX11 | -1.11 | 12p11.21 | regulate transcription, splicing, translation,  and DNA repair, participate cell cycle |
| DNM2 | -1.04 | 19p13.2 | promote apotosis, promote cell cycle progression |
| E2F4 | -1.37 | 16q22.1 | regulate cell cycle progression |
| GAPDH | -1.09 | 12p13.31 | glycometabolism |
| GTF2H1 | -1.14 | 11p15.1 | promote cell cycle progression,  participate DNA repair |
| HERC5 | -1.06 | 4q22.1 | promote cell cycle progression |
| HUS1 | -1.15 | 7p12.3 | participate DNA repair, regulate cell cycle |
| MAD2L1 | -1.02 | 4q27 | promote cell cycle and mitosis |
| MAD2L2 | -1.11 | 1p36.22 | promote cell cycle and mitosis |
| MCM2 | -1.01 | 3q21.3 | participate cell cycel, DNA replication,  and cell division |
| MKI67 | -1.15 | 10q26.2 | promote cell cycle progression,  cell proliferation/growth |
| MNAT1 | -1.07 | 14q23.1 | promote cell cycle progression, cell proliferation, participate DNA repair |
| MRE11A | -1.13 | 11q21 | regulate mitosis, promote proliferation |
| PCNA | -1.09 | 20p13-p12 | promote cell cycle progression, cell proliferation, participate DNA replication and repair |
| RBBP8 | -1.09 | 18q11.2 | regulate cell cycle |
| RPA3 | -1.03 | 7p21.3 | participate DNA replication |
| RPL13A | -1.01 | 19q13.3 | protein metabolism, promotes proliferation |
| SERTAD1 | -1.44 | 19q13.1-q13.2 | promote cell proliferation |
| TFDP1 | -1.10 | 13q34 | promote cell cycle progression,  and cell proliferation |
| TFDP2 | -1.04 | 3q23 | regulate cell cycle |
| UBA1 | -1.07 | Xp11.23 | initiate the activation and  conjugation of ubiquitin-like proteins |
