# Supplementary material for: PinX1 suppresses bladder urothelial carcinoma cell proliferation via the inhibition of telomerase activity and p16/cyclin D1 pathway
Source: Mol Cancer. 2013 Nov 23;12:148. doi: 10.1186/1476-4598-12-148 (PMC4176126; doi:10.1186/1476-4598-12-148)
Supplement: Additional file 3: Table S2 — Association between expression of PinX1 and p16 and cyclin D1 in UCB. [file 1476-4598-12-148-S3.doc]

| **Table S2** Association between expression of PinX1 and p16 and cyclin D1 in UCB | | | | |
| --- | --- | --- | --- | --- |
|  |  | PinX1 protein | |  |
| Variable | Cases | Negative expression | Positive expression | *P*value^a^ |
| p16 |  |  |  |  |
| Negative expression | 97 | 54(55.7%) | 43(44.3%) | 0.001 |
| Positive expression | 90 | 29(32.2%) | 61(67.8%) |  |
| cyclin D1 |  |  |  |  |
| Negative expression | 85 | 12(14.1%) | 73(85.9%) | <0.001 |
| Positive expression | 102 | 71(69.6%) | 31(30.4%) |  |
| ^a^Fisher’s exact test. UCB: urothelial carcinoma of bladder. | | | | |
